# Determination of blue-light-induced infrared absorption based on mode-matching efficiency in an optical parametric oscillator

**DOI:** 10.1038/srep41405

**Published:** 2017-02-01

**Authors:** Yajun Wang, Wenhai Yang, Zhixiu Li, Yaohui Zheng

**Affiliations:** 1 The State Key Laboratory of Quantum Optics and Quantum Optics Devices, Institute of Opto-Electronics, Shanxi University, Taiyuan 030006, China; 2 Collaborative Innovation Center of Extreme Optics, Shanxi University, Taiyuan, Shanxi 030006, China

## Abstract

Non-classical squeezed states of light at a compatible atomic wavelength have a potential application in quantum information protocols for quantum states delaying or storaging. An optical parametric oscillator (OPO) with periodically poled potassium titanyl phosphate (PPKTP) is the most effective method for generating this squeezed state. However, it is a challege for the nonlinear interaction in PPKTP crystal at the D1 line of rubidium atomic, due to a strong blue-light-induced infrared absorption (BLIIRA). In this paper, we report an indirect measurement method for the BLIIRA through measuring the mode-matching efficiency in an optical parametric oscillator. In contrast to previous works, our method is not limited by the absolute power variation induced from the change of frequency conversion loss and the impedance matching originated from the change of absorption loss. Therefore, the measurement process is performed at the phase-matching condition. The measured results show that BLIIRA coefficient is quadratic dependence of blue light intensity below 1 kW per square centimeter in our PPKTP device, which will provide important basis for optimizing squeezed state generation at 795 nm.

Exceptionally high nonlinearity and good flexibility makes periodically poled potassium titanyl phosphate (PPKTP) crystal an attractive material candidate for frequency doubling^1^,^2^,^3^ as well as for the reverse process of parametric oscillation^4^,^5^,^6^,^7^,^8^ in continuous wave (CW) or pulse wave (PW). For CW operation, the latter process is the most effective method for generating squeezed state with the highest degree of quadrature phase squeezing^5^,^6^,^7^,^8^. The nonlinear crystal is placed inside a single resonant optical cavity defined as a squeezing resonator. The squeezing factor is ultimately limited by optical losses for the generation of high-level squeezed state^9^. The optical loss has direct relationship with the cavity’s round-trip loss, which is partly determined by the absorption loss of the nonlinear crystal.

Low passive losses of PPKTP are of high interest for the efficient generation of strongly squeezed state resonant on the ^87^Rb D1 line (795 nm)^10^. However, in the experiments of squeezed state generation, the second harmonic beam at 397.5 nm (blue-UV range) is used as a pump light, which would induce color centers to increase the near infrared (IR) absorption in the crystal. This phenomenon is called blue-light-induced infrared absorption (BLIIRA)^11^,^12^,^13^,^14^,^15^,^16^, an effect of increased IR absorption in the presence of blue light. The color centers are associated with Ti^4+^/Ti^3+^ electron traps, O^2−^/O^−^ hole traps, K^+^ and V(K^+^) traps of free carriers or Fe^3+^ hole traps, which come from the native stoichiometry defects or impurities of the cystal^14^,^16^. Based on the nature of color centers, the BLIIRA can be explained by two-center charge-transport model^11^: the traps, between the conduction and valence bands, are divided into deep one and shallow one. Without 397.5 nm laser illuminating, the IR photons can not interact with the charges in deep traps, and the shallow traps are empty. With 397.5 nm laser illuminating, the charges in the deep trap fall into the shallow trap via the valence band, which will induce the interaction between IR photons and charges in the shallow traps. The traps concentration depends strongly on the crystalline growth condtion and quality, hence should be solely considered from sample to sample. Therefore, the absorption loss in PPKTP includes not only passive loss, but also additional loss induced by BLIIRA. BLIIRA loss is a serious limitation for the quantum noise reduction because of the additional absorption loss and non-optimal mode-matching efficiency arising from the thermal effect^9^,^17^. The squeezing factor increases with the second harmonic pump intensity, however, the pump intensity does also increase BLIIRA loss, which is adverse to the squeezing factor. There is a contradiction between increasing pump intensity and reducing BLIIRA loss. In order to obtain the optimal squeezing factor, it becomes urgent to find the optimum compromise between the harmonic pump intensity and BLIIRA loss. Additionally both parameters are sensitive to the crystal length and the cavity mode size, which makes it too complex to optimize the squeezing factor experimentally. It is thus necessary to investigate the relationship between pump intensity and BLIIRA loss experimentally in order to optimize the squeezing factor.

BLIIRA in KNbO_3_ crystal had been studied in detail. In refs 10, 11, it was measured by placing the KNbO_3_ in a single resonant optical cavity (SROC), and then comparing the IR transmission of the SROC on resonance with and without blue light injected. In ref. 12, two beams collinearly propagate through the crystal to observe the additional temperature variation with and without blue light, and then BLIIRA can be calculated. In refs 13, 14, 15, the green-light induced IR absorption (GLIIRA) or BLIIRA in KTP and PPKTP were measured by using the He-Ne laser as a probe beam, then GLIIRA (or BLIIRA) can be obtained by phase distortion of the probe beam. They had given a clear demonstrating of the remnant absorption relaxation process, the temperature-dependent and blue/green intensity-dependent rules for different isomorphs. However, in these works described above, IR and blue light with random wavelengths do not satisfy frequency doubling condition, which is inconsistent with the relationship between pump and signal beams in an optical parametric oscillator (OPO). Therefore, these results can not characterize the BLIIRA loss in OPO.

In this work, we report on a method for the BLIIRA measurement based on a SROC, by comparing the difference of mode-matching efficiency (MME, *κ*_00_) in a SROC with and without blue light. The basis of the proposed mechanism is as follows: the eigenmode size of the SROC changes with the thermal focal length arising from the absorption, which alters the MME between the seed beam and the SROC’s eigenmode. Thus, the absorption can be quantified by the MME. According to the operating condition of squeezing resonator at 795 nm, we obtain the BLIIRA with both the fundamental and harmonic waves present in PPKTP under phase-matching condition. The results show that the BLIIRA coefficient is quadratic dependence of the blue light intensity below 1 kW/cm^2^. Subsequently, we do also compare the measured results of MME deviation with the non-phase-matching one. Due to the influence of nonlinear optical process, the result under phase-matching is a little higher than that of the non-phase-matching. This result is important to find the trade-off between pump intensity and BLIIRA loss, and obtain the best squeezing factor at 795 nm.

## Results

### The basic principle of BLIIRA measurement

**Figure 1:**
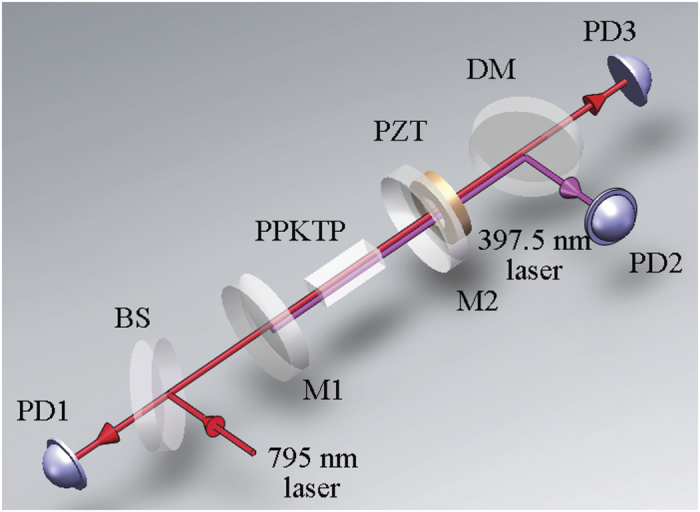
Experimental configuration of the BLIIRA measurement. PD 1–3: Photodiode detector 1–3; BS: 50/50 beam splitter; DM: dichroic mirror; M 1–2: concave mirror 1–2; PZT: Piezoelectric transducers.

Figure 1 shows the experimental configuration for absorption measurement of PPKTP crystal. The laser source is a home-made CW Ti:sapphire laser with a maximum output power of 1.27 W^18^, and can be finely tuned around the wavelength of 795 nm corresponding to the transition of ^87^Rb D1 line. A CW single frequency 532 nm laser is used as the pump source with the maximum output power of 18 W^19^,^20^. A part of the 795 nm laser is injected into a four-mirror enhanced-external-cavity for CW second harmonic generation (SHG), which is used as a laser source for the absorption measurement of 397.5 nm laser and the detailed SHG progress can be found in ref. 3. The remaining part of it is entered into the SROC for BLIIRA measurement. Both the 795 nm and 397.5 nm lasers operate in fundamental transverse and single longitudinal mode during the measurement process to avoid the influence of high order modes on MME measurement. The SROC consists of two concave mirrors (M1 and M2) with curvature radius of 30 mm and a PPKTP crystal. The distance between them is 57.5 mm, which forms a waist radius of 38.9 *μ*m at 795 nm or 27.6 *μ*m at 397.5 nm, which is analogue to the OPO in our squeezed state generator. The concave end face of M1 (or M2) is coated with high reflectivity (HR) at 397.5 nm and partly transmission T_795_ = 3.6% at 795 nm (or HR795 nm and high transmission (HT) at 397.5 nm), and the plane face is anti-reflecting (AR) coated at both wavelengths. M1 plays a role as an input coupler of 795 nm beam, and M2 acts as the output coupler of 795 nm and 397.5 nm beams. The dimension of the flux-grown PPKTP crystal (Raicol Crystals) is 1 × 2 × 10 mm^3^ and the two end faces are both coated with AR795/397.5 nm, which is periodically poled using micro lithographic techniques^21^, but we have limited knowledge for the growth condition and process of the crystal. The crystal is a-cut with the c axis perpendicular to the cross section of 2 × 10 mm^2^. The crystal is placed into a copper oven for temperature control, and the oven is thermally insulated from the ambient by a polyarylsulfone layer, which is fixed on an xyz translation stage. The temperature range of the oven is 40–160 °C. The periodically poled of the PPKTP is 3.15 *μ*m, with the phase-matching (PM) temperature and temperature acceptance bandwidth of 55 °C and 1.1 °C, respectively. PD2 detects the transmission power of 397.5 nm laser exporting from M2, and the circulating power in SROC can be inferred from the dichroic mirror (DM) and M2 transmittance and the measured values of PD2. PD3 is used for recording the MME of the cavity and reading the error signal for Pound-Drever-Hall technique^22^. The 50/50 beam splitter (BS) separates part of the reflected 795 nm beam of M1 to detect its circulating power in SROC by PD1. The dichroic mirror (DM) is coated with HT795 nm/HR397.5 nm and is used to separate the 795 nm laser from the 397.5 nm one.

Before measuring the BLIIRA, a beam at 397.5 nm from the enhanced-external-cavity propagates directly through the PPKTP crystal without SROC, whose temperature is set as 70 °C and its polarization direction is parallel to the c axis of the crystal, which is in accordance with the PM condition. The beam radius is mode-matched to 27.6 *μ*m at the center of PPKTP, which is identical to that used for BLIIRA measurement. The mode size was checked by an optical beam profiler (BP209-VIS, Thorlabs). The absorption coefficient at 397.5 nm (*α*_*Blue*_ ≈ 18.6%/cm) is calculated from the ratio of optical power after and before the PPKTP crystal. The optical power is measured by photodetector with good linearity. The measured result is an average value for 10 times measurements and is close to the data in ref. 15, but has minor difference, which is attributed to the different growth conditions from one sample to another^11^,^16^,^23^,^24^. With the change of laser power at 397.5 nm, no significant change of the absorption coefficient is observed. The PPKTP crystal has low passive absorption at 795 nm (less than 150 ppm/cm), which can be neglected^3^,^25^.

Secondly, with the same experimental condition and method above, a 397.5 nm and a 795 nm lasers are combined with a DM and collinearly propagate through the center of the crystal, which are focused to the waist radius of 38.9 *μ*m and 27.6 *μ*m with a telescope system, respectively, and the temperature of the cystal is adjusted to 70 °C to aviod the influence of nonlinear interaction process for absorption measurement. The mode sizes are also confirmed by the optical beam profiler. The laser power at 397.5 nm ranges from 0 to 40 mW (corresponding to a maximum power intensity of 1.36 × 10^3^ W/cm^2^), and that of 795 nm is between 0 and 500 mW. For a certain blue beam intensity, e.g. 40 mW, we found that the IR absorption is always a constant with the IR power increasing. This result confirms that the IR absorption is independent of the IR power, and only dependent of the intensity of 397.5 nm laser. This procedure is only used to check the dependence of 795 nm beam absorption on IR intensity for a certain blue power, but can not obtain the exact values of IR absorption.

Finally, to analyze the total absorption feature of the PPKTP crystal with BLIIRA, we perform the experiment under PM and NPM with configuration in Fig. 1 by measuring the MME of the SROC^3^,^26^, respectively. The MME represents the spatial mode overlap efficiency between the fundamental mode of the injected IR beam and the TEM_00_ eigenmode of the SROC. The principle of the absorption measurement is as follows. The absorption process of PPKTP is associated with the generation of heat. The combination of volumetric heating and surface temperature controlling leads to a temperature gradient and forms a thermal lens in the PPKTP crystal^27^,^28^. Thermal lens alters the eigenmode size of SROC, which deteriorates MME between IR beam mode and the cavity mode^3^. Thus, the relationship between the deviation of MME (Δ = 1 − *κ*_00_) and the IR absorption coefficient *α*_*IR*_ is established by two intermediate quantities (thermal lens and cavity eigenmode). Then, we can begin the absorption measurement with the total thermal lens *f*_*total*_, which is the combined action of IR and blue light absorption, and can be expressed to refs 27, 28,


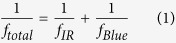



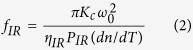


where *f*_*IR*_ is TFL induced by infrared absorption, and *f*_*Blue*_ is TFL due to blue absorption which has a common expression with *f*_*IR*_; *ω*_0_ and *ω*_0*Blue*_ are the waist radius of IR and blue light in the PPKTP crystal, respectively; *P*_*IR*_ and *P*_*Blue*_ are the circulating powers of IR and blue light in the cavity^3^, which can be inferred experimentally from the measured value of PD1 and PD2, respectively; *η*_*IR*_ and *η*_*Blue*_ are the absorption efficiencies of IR and blue light (*η* = 1 − *e*^−*αl*^), respectively; *l, K*_*c*_ and *dn*/*dT* are the length, thermal conductivity and thermo-optical coeffecient of the crystal, respectively. If the total and blue beam induced thermal lenses are known, we can obtain the thermal lens of IR light. Then, for IR beam, expression (2) can be transferred into,


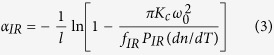


For an OPO similar to Fig. 1, there are three optical elements (M1, M2, PPKTP) in the cavity. Based on ABCD matrix method, the total TFL *f*_*total*_ can be referred from the cavity eigenmode size. In our SROC, the IR beam oscillates in the optical cavity, which can be used to observe the MME between the injected IR beam and the cavity eigenmode. During the measurement process, the mode size of the injected IR beam remains constant, and the MME is only dependent of the cavity eigenmode size. The MME ultimately infers the total thermal lens. The eigenmode waist radius of IR laser *ω*_0*e*_ relates to the MME (*κ*_00_) according to the expression below^3^,^26^,


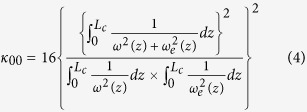



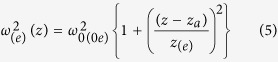


where *L*_*c*_ is the cavity length; *ω(z*) and *ω*_*e*_(*z*) are the beam radius of the incident light and cavity eigenmode at the position of *z*, respectively; *ω*_0_ (a constant waist radius of 38.9 *μ*m) and *ω*_0*e*_ are the waist radius of IR beam and cavity eigenmode; *z*_*α*_ is the beam waist position; 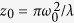, 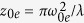. In a word, *f*_*total*_ can been deduced from the measured value of MME (*κ*_00_ → *ω*_0*e*_ → *f*_*total*_); Subsequently, we can extract the IR thermal lens *f*_*IR*_ from *f*_*total*_ with formula (1) and (2), the measured blue power, blue light absorption coefficient and other constants in the equations; At last, *α*_*IR*_ is figured out by formula (3) (*f*_*IR*_, *ω*_0_ → *α*_*IR*_). Due to the lower linear absorption of 795 nm laser^25^, *α*_*IR*_ is approximately equal to BLIIRA coefficient, i.e., *α*_*BLIIRA*_ ≈ *α*_*IR*_.

**Figure 2:**
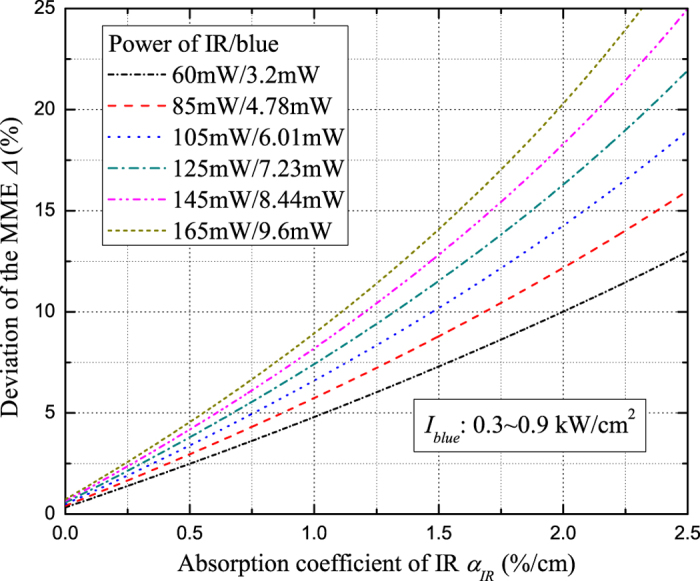
MME deviation Δ versus the absorption coefficient of IR *α*_*IR*_ at different incident powers.

Based on the above analysis, we can establish the relationship between IR absorption and MME with the formulas (1)–(5), which is shown in Fig. 2. In the analysis procedures, the TFL of blue light is experimentally inferred from the absorption coefficient (a measured value of *α*_*Blue*_ ≈ 18.6%/cm) and the SHG circulating power (inferring from the detected value of PD2) in the crystal with formula (2). With the increasing of IR power, the blue light is generated gradually (the optical power (intensity) ranges from 3.2 mW (0.3 kW/cm^2^) to 9.6 mW (0.9 kW/cm^2^)). As shown in Fig. 2, if BLIIRA is present at a certain input power, it will give rise to more absorption of IR and more deviation of MME. Therefore, we can deduce the IR absorption coefficient based on the deviation of MME from the initial value. It is worth mentioning that MME is a ratio of the TEM_00_ eigenmode power and the total eigenmodes power of the oscillating modes in the cavity^26^, which is mainly influenced by the TFL induced TEM_00_ eigenmode evolution. The nonlinear process makes the thermal gradient deviate from theoretical analization along the a-axis of the crystal, which would weakly affect the measured results.

### Experimental results of BLIIRA

We bought the three PPKTP samples of the same batch from the Raicol Crystal Ltd, and all of them were not used in any experiments beforehand. One of the crystals is randomly selected for SHG and the result is shown in ref. 3, which is prepared for the absorption measurement of blue light. By carefully moving the PPKTP crystal over the aperture of 1 × 2 mm^2^ the output power of the SHG is no obvious changes, which shows that the crystal’s feature is uniform.

The BLIIRA measurement is performed with the other two samples by using the configuration in Fig. 1. The measurement process is as follows: Firstly, a 795 nm laser of approximately 1 mW in pure TEM_00_ mode, with the polarization direction parallel to the PM direction, is aligned and mode-matched to the SROC. The low power level induces little heat contribution to the material, and we assume that the mode size of the injected IR beam is approximately equal to the initial TEM_00_ eigenmode of the SROC. The mode-matching efficiency, between 795 nm laser mode and the TEM_00_ eigenmode of the SROC, can be obtained by the intensity ratio of the main transmission peak to the whole transmission peaks among a free spectral range (FSR) of the SROC. The initial mode-matching efficiency (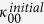) is optimized to 99.2%. The intensity of the transmission peaks is recorded by an oscilloscope using PD3. Subsequently, the PPKTP temperature is tuned to 70 °C deviating from PM condition, and the input IR power is increased to 60 mW with no obvious blue laser generated. We record the MME (under non-phase-matching (NPM) at 60 mW input power, 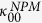) from the acquired data of the oscilloscope. Thirdly, the temperature is tuned to 55 °C to satisfy the PM condition. When the optical cavity length is locked on the laser frequency by an electronic feedback circuit, the blue laser is continuously exported, whose intensity is inferred from the detected power of PD2. The operating time for the cavity locking should be more than the BLIIRA build-up^15^, until the detected power of PD2 achieves the stable value. After 20 min, the cavity is unlocked and scanned, and we immediately record the transmission peaks by PD3 within the BLIIRA relaxation time^15^ to capture the MME (under PM at 60 mW input power, 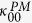). At last, according to the deviation value 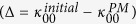 of the MME, the BLIIRA at this power can be obtained from the results of Fig. 2. In order to remove the remnant BLIIRA, the PPKTP temperature is raised gradually to 155 °C before the next BLIIRA measurement. Then, we observe the MME in real-time, till the MME returns to 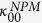, which is too important to overcome the influence of the remnant BLIIRA on the following measurements. It is worth mentioning that the relaxation time of the remnant BLIIRA can be shortened by increasing the PPKTP temperature, as a result of reducing the charges concentration of long-lived color centers^16^. The operating steps are repeated over again at each power point, the one-to-one correspondence relationship between the BLIIRA and laser power at 397.5 nm is built. With the results of 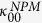 at each power levels, we can also obtain the linear absorption coefficient of IR light without blue beam, which is lower than 150 ppm/cm, and the sensitivity of the measuring device is evaluated to be better than 10^−5^/cm. Limited by the real-time recording capacity of our method, it is unable for us to provide the exact BLIIRA buid-up and relaxation time.

**Figure 3:**
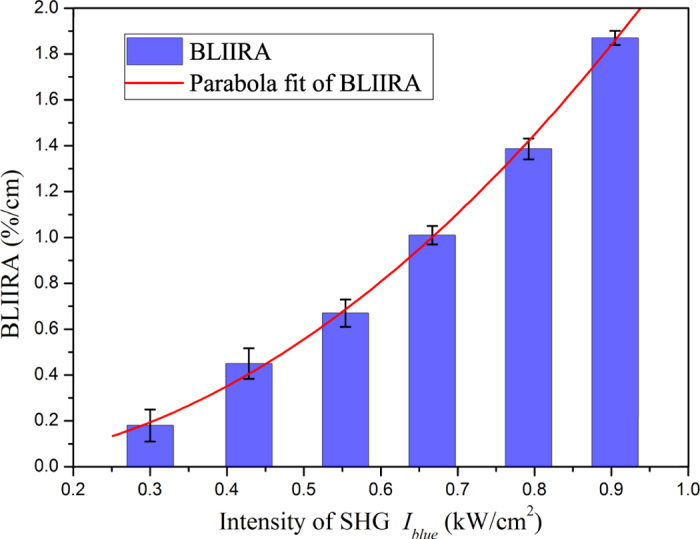
BLIIRA coefficient *α*_*BLIIRA*_ as a function of the intensity of 397.5 nm laser *I*_*blue*_; the data is fitted with 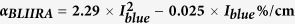 (*I*_*blue*_: kW/cm^2^).

**Figure 4:**
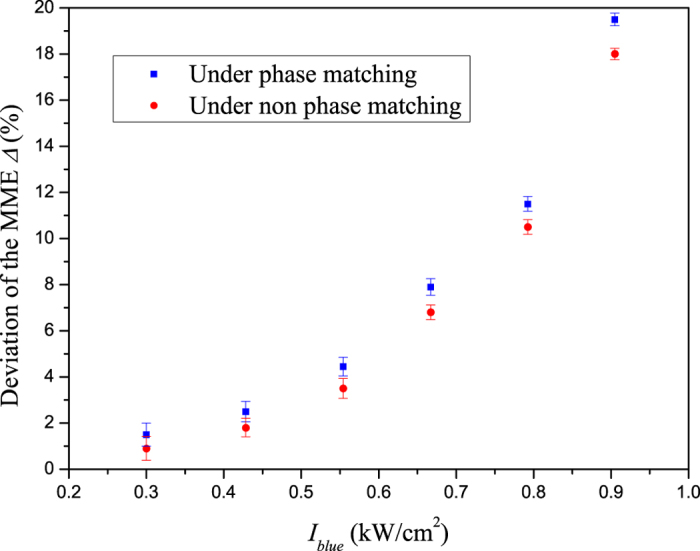
The results of MME deviation under phase and non-phase-matching conditions.

The two samples are adopted in turns to investigate the BLIIRA. For each sample, the BLIIRA measurements were repeated 10 times at each IR power to reduce the experimental error. The BLIIRA of the two samples has a tiny difference, hence we only show the results for one of them in Fig. 3 (column diagram). At very onset, the MME of the resonator is 99.2% with 1 mW IR input power under PM condition. With the increase of IR input power, the MME gradually decreases. To observe remarkable MME variation, the IR input power is increased from 60 mW to 165 mW, and the MME of the cavity decreases from 98.8% to 81.5%, which are shown using the blue squares in Fig. 4. Based on the detected power of PD1 (or PD2) and mirrors transmittance, the corresponding intensity in the crystal at 795 nm (or 397.5 nm) is obtained in the range of 1.26 kW/cm^2^ to 3.47 kW/cm^2^ (or 0.3 kW/cm^2^ to 0.9 kW/cm^2^). By excluding the absorption of blue light contribution to MME, the BLIIRA absorption *α*_*BLIIRA*_ can be figured out by the experimental data with the results of Fig. 2, which lies between 0.18%/cm and 1.87%/cm. We estimate that the overall system uncertainty is about 10%, which arises from the BLIIRA relaxation after unlock, the measurement error of MMEs, the light intensity, the original passive loss of IR and blue light. Inferring from the measurement results in Fig. 3, the variation tendency of the IR absorption is supported by the assumption of a quadratic dependence of green/blue light intensity^29^,^30^. BLIIRA coefficient can be fitted with the expression 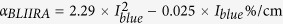 (*I*_*blue*_: kW/cm^2^) below 1 kW/cm^2^. Due to the different absorption properties from sample to sample, this result is only responsible for the current PPKTP and power levels. The measurement result is helpful to optimize the pump intensity and generate high-level squeezed state with this crystal, in an actual OPO for squeezed state generation at 795 nm. The approximately quadratic dependence of blue light intensity can be explained by the two-center charge-transport model: the stronger blue light intensity increases the concentration of holes in the valence band, which increases the interaction possibility between shallow traps and IR photons^11^. The strong BLIIRA in this PPKTP is attributed to the shorter wavelength^13^, larger ionic conductivity in KTP^16^ and ferroelectric domain inversion induced spatial redistribution of the K^+^ and V(K^+^)^14^. Since the MME intensely changes at higher harmonic wave power density, the measurement result is more sensitive at a higher SHG intensity, which is verified by the error bars in Fig. 3. Limited by the quality of the PPKTP, increasing further the blue light intensity, the strong photoionization induces in the occurrence of gray-tracking, which affects the measured results of BLIIRA loss.

In order to experimentally quantify the influence of the nonlinear process on the measurement results, we also measured the MME deviation under NPM condition. The temperature of the crystal is tunned to 70 °C, which is far away from the PM temperature. Both the fundamental and harmonic waves are mode-matched into the SROC. Except for the operating temperature of the sample, the other conditions of the measurement are the same as the former measurement process. The result is shown in Fig. 4 with red circles, and the corresponding MME deviation under PM condition is also presented with blue squares. Knowing from Fig. 4, the measured values under PM condition are a little higher than that of under NPM condition. Moreover, the difference between them increases with the increase of the harmonic wave intensity. The phenomenon can be attributed to the absorption gradient along the a-axis, originating from nonlinear interaction process.

## Discussion

In summary, the BLIIRA coefficient in PPKTP under PM condition is reported here for the first time to our knowledge, with the fundamental and harmonic waves passing through the crystal simultaneously. The absorption loss is calculated through the change of MME in a SROC, which arises from the thermal lens induced absorption heating. The measured results show that BLIIRA coefficient is quadratic dependence of blue light intensity below 1 kW/cm^2^. Differing from other works by comparing the absolute transmission power after and before blue laser injected, this method is not limited by absolute power variation induced from the change of frequency conversion loss and impedance matching originated from the variation of absorption loss. The measurement is performed under PM condition, with two beams satisfying frequency doubling relationship, which is completely similar to the operating condition of OPO. Subsequently, the MME under NPM condition is also measured to quantify the influence of nonlinear process. Comparing with the measured value under NPM condition, the measured deviation of MME under PM condition is a little higher, which can be explained as the absorption gradient along the optical a-axis, coming from nonlinear interaction process.

The results will spur us to find an optimum compromise between the harmonic pump intensity and BLIIRA loss to provide important basis for the optimizing of squeezing resonator. However, the method is only suitable to measure the BLIIRA in moderate blue light intensity. For high blue light intensity, severe thermal effect introduces transmission peaks distortion of OPO to increase the measurement error of MME, which increases the measured uncertainty. We hope to overcome the intensity limitation for satisfying much more possible applications.

## Additional Information

**How to cite this article**: Wang, Y. *et al*. Determination of blue-light-induced infrared absorption based on mode-matching efficiency in an optical parametric oscillator. *Sci. Rep.* **7**, 41405; doi: 10.1038/srep41405 (2017).

**Publisher's note:** Springer Nature remains neutral with regard to jurisdictional claims in published maps and institutional affiliations.
